# A Case-Control Study on Risk Factors for Preterm Deliveries in a Secondary Care Hospital, Southern India

**DOI:** 10.1155/2014/935982

**Published:** 2014-03-13

**Authors:** Chythra R. Rao, Lara E. E. de Ruiter, Parvati Bhat, Veena Kamath, Asha Kamath, Vinod Bhat

**Affiliations:** ^1^Department of Community Medicine, Kasturba Medical College, Manipal University, Manipal, Karnataka 576104, India; ^2^University Medical Center Groningen (UMCG), P.O. Box 30.001, 9700 RB Groningen, The Netherlands; ^3^Dr. TMA Pai Hospital, Manipal University, Udupi, Karnataka 576101, Udupi, India

## Abstract

*Introduction.* Preterm birth is the leading cause of newborn deaths and the second leading cause of death in children under five years old. Three-quarters of them could be saved with current, cost-effective interventions. The aim of this study was to identify the risk factors of preterm birth in a secondary care hospital in Southern India. *Methods.* In the case-control study, records of 153 antenatal women with preterm birth were included as cases. Age matched controls were women who had a live birth after 37 weeks of gestational age. Gestational age at delivery and associated risk factors were analyzed. *Results.* The preterm birth rate was 5.8%. Common risk factors associated with preterm birth were hypertensive disorders of pregnancy (21.4%), height <1.50 m (16.8%), premature rupture of membranes (17.5%), and fetal distress (14.9%). Mean birth weight for preterm babies was 2452 grams while the birth weight for term babies was 2978 grams. *Conclusion.* The commonest obstetrical risk factor for preterm birth was hypertensive disorders of pregnancy and nonobstetrical risk factor was height <1.50 m. The percentage of preterm birth was low, comparable to developing countries.

## 1. Introduction

Preterm birth (PTB) is the leading cause of infant morbidity and mortality in the world. The World Health Organization (WHO) defines preterm birth as any birth before 37 completed weeks of gestation or fewer than 259 days since the first day of woman's last menstrual period (LMP).

In developing countries, the main causes of preterm births include infectious diseases and poor availability and accessibility of health care resources. In high-income countries, the increase in the number of preterm births is linked to conception among older women and increased number of multiple pregnancies as a result of usage of fertility drugs. In some developed countries, medically unnecessary inductions and caesarean section deliveries before full term also increase preterm birth rates. In rich and poor countries, many preterm births remain unexplained [1, 2].

Approximately three-fourths of perinatal deaths occur in foetuses that are delivered at <37 weeks, and about 40% of these deaths occur in those delivered at <32 weeks. In addition to its contribution to mortality, preterm birth has lifelong effects on neurodevelopmental functioning such as increased risk of cerebral palsy, impaired learning, and visual disorders and an increased risk of chronic disease in adulthood [3]. The economic cost of preterm birth is high in terms of neonatal intensive care and ongoing health care and educational needs. The social cost is also high, with many families experiencing the sudden loss of a preterm baby or a stressful hospital stay, sometimes for months [2].

Defining risk factors for prediction of preterm birth is a reasonable goal for several reasons. First, identification of at-risk women allows initiation of risk-specific treatment. Second, the risk factors might define a population useful for studying specific interventions. Finally, identification of risk factors might provide important insights into mechanisms leading to preterm birth. Yet, data regarding preterm births and risk factors are not routinely collected in hospitals. Therefore, to obtain insight into the risk factors for PTB, a secondary care hospital was chosen in Southern India for the study.

## 2. Materials and Methods

Data of births in Dr. TMA Pai Hospital, Udupi, during the period from January 2010 to May 2013 were used for this study. Approval for the study was taken from the Institutional Ethics Committee. The births at Dr. TMA Pai Hospital are registered in a log book in the labour room. All live births during the period from January 2010 to May 2013 were included into this study. Potential cases were all women who were recorded in the labour log book as giving birth at a gestational age between 28 and 37 weeks. The hospital numbers of these women were taken from the log book and the patient records were collected from the hospital medical records. Data from these records were used to fill the questionnaire.

For every case record, two control records were obtained. Controls were all women who had a live birth after 37 weeks of gestational age. The controls were age matched. For every case, two controls two years older or younger than the case were selected by simple random selection. This selection was done by using a random number table.

There were in total 238 cases of preterm births during the study period. Out of which 17 babies were still born, 47 records were not found, and 20 cases had inadequate data and hence were excluded from the study so the final analysis was done for 154 cases.

The gestational age was assessed by using date of last menstrual period and confirmed by ultrasound in the records. The criteria used for defining the different clinical conditions are shown in Table 1.

The questionnaire used for data entry was divided into six sections: “background information of the mother,” “medical history,” “current pregnancy details,” “baby details,” “details of previous conceptions,” “medical disorders complicating current pregnancy,” and “investigations.” In the “background information of the mother” section, information was obtained about the age, occupation, height, and the body weight at the beginning of the pregnancy. In the “medical history” section, information was obtained about family and medical history of the pregnant woman.

Risk factors found in the women were categorized into twelve categories: “fetal distress”; “failed induction”; “hypertensive disorders of pregnancy”; “malpresentation and multiple pregnancy”; “previous uterine scar”; “antepartum haemorrhage (APH)”; “PROM”; “medical disorders”: anemia, hyperthyroidism, hypothyroidism, Rh negative with ICT positive, and Rh negative; “oligo/polyhydramnios”; “gestational diabetes mellitus (GDM)”; “cephalopelvic disproportion”; and “others”: manual removal of placenta, baby hydrocephalus with absent posterior vault, bladder injury, and fetal polycystic kidneys.

Univariate logistic regression analysis was used to examine the association between preterm birth and risk factors. The univariate association of risk factors with preterm birth was approximated by determining the odds ratio. The statistical package IBM SPSS Statistics 20.0 was used for all statistical calculations. Odds ratio was calculated for the risk factors associated with preterm and term deliveries. Univariate and multivariate logistic regression analysis were used to examine association between risk factors and preterm and term birth. Statistical significance was set at 5% level. Ninety-five percent confidence intervals for the odds ratio were calculated.

## 3. Results

Among the 4,137 antenatal admissions during the study period, 238 were admitted with preterm labour. The preterm birth percentage was 5.8%.

The baseline characteristics are described in Table 2. The mean birth weight of the 167 (33.1%) preterm babies was 2452.7 grams and the mean birth weight of 338 (66.9%) term babies was 2977.8 grams.

Table 3 shows that the most common mode of delivery was spontaneous vaginal delivery, respectively, 41.4% and 51.2% for cases and controls. Emergency lower segment caesarean section (LSCS) occurred more in the preterm group than in the control group.

The mothers in both the case and control group were healthy, with very few preexisting medical conditions as shown in Table 4. The most common antenatal complication was pregnancy induced hypertension (OR = 4.5, 95% CI 1.34–15.25).

Risk factors associated with preterm delivery using univariate analysis are presented in Table 5. Hypertensive disorders of pregnancy were found to be the most common cause of preterm labour (21.4%). Height less than 1.50 m as the common risk factor was seen in 26 (16.8%) women. Premature rupture of membranes was found in 27 (17.5%) women. Fetal distress occurred among 23 (14.9%) women and oligo- or polyhydramnios in 19 (12.3%) women.

In the control group the most common complication was fetal distress, found in 53 (15.9%) women, followed by failed induction 45 (13.5%), GDM 27 (8.1%), and previous uterine scar 27 (8.1%).

Significant associations were found between hypertensive disorders of pregnancy, height <1.50 m, PROM, oligo-/polyhydramnios, threatened abortion, twin gestation, and preterm birth.

## 4. Discussion

The present study has shown that preterm delivery was significantly associated with hypertensive disorders of gestation, height <1.50 m, PROM, oligo-/polyhydramnios, threatened abortion, and twin gestation.

Hypertensive disorders of pregnancy were present in 21.4%, which were the commonest obstetrical risk factor. The finding was contradictory to the study done by Shresta et al. [3] in which hypertensive disorders of pregnancy were seen in 13.3%. The commonest obstetrical risk factor in that study was APH (23.3%).

Analysis of nonobstetrical risk factors revealed height <1.50 m as a significant risk factor for preterm birth. Short women are more likely to have a small pelvis, which can lead to obstructed labour. Intrauterine growth restriction is also more likely.

In late pregnancy, placenta previa and placental abruption are often associated with vaginal bleeding and often lead to preterm birth (PTB) [4]. In this study, PROM was seen in 27 (17.5%) and 21 controls (6.3%).

Multiple gestations, accounting for only 2-3% of infants, carry a substantial risk of preterm delivery and result in 15–20% of all preterm births. Nearly 60% of twins are born preterm. About 40% of twins will have spontaneous labour or PROM before 37-week gestation, with others having an indicated preterm delivery because of preeclampsia or other maternal or fetal disorders [5]. The widespread availability of assisted reproduction has resulted in a large increase in the incidence of multiple gestations and this increase, in turn, has led to an increase in the preterm birth rate [5]. The mechanism for preterm labour in multiple gestations and particularly higher order multiple gestations may be related to uterine distension, increased intrauterine volume, or related complications such as cervical incompetence. In particular, higher circulating levels of relaxin associated with superovulation may cause cervical insufficiency, with subsequent PTB [6]. Reduction of multifetal gestations, particularly high order multifetal gestations, may improve neonatal outcome.

According to Krupa et al. [7] vaginal bleeding caused by placental abruption or placenta previa is associated with a very high risk of preterm delivery, but bleeding in the first and second trimesters that is not associated with either placental abruption or placenta previa is also associated with subsequent preterm birth. The present study did not find any significant association between APH and preterm birth.

Extremes in the volume of amniotic fluid—oligo- or polyhydramnios—are associated with preterm labour [8], although the association was not statistically significant in the present study.

Maternal demographic characteristics associated with preterm birth include low socioeconomic and educational status, low and high maternal ages, and single marital status [9, 10]. Observational studies of the type of work and physical activity related to preterm birth have produced conflicting results. The level of physical activity is not consistently related to the rate of preterm birth [11–14]. The limitation of the current study was inability to assess these nonobstetrical risk factors like socioeconomic status, maternal malnutrition, cigarette smoking, and direct abdominal trauma as these data were not found in the records, but all the women were married as marriage is the usual norm of the society.

Based on data from 184 countries, the global average preterm birth rate in 2010 was 11.1%. Preterm birth rates varied widely between countries. At a national level, the estimated preterm birth rate ranged from about 5% to 18%. The highest rates of preterm birth were in south-eastern Asia and sub-Saharan Africa (13.5% and 12.3% of all livebirths, resp.). Studies revealed incidence to be higher in developing countries than in developed countries [15]. India has the highest preterm birth rate; incidence of around 13.0% [2] has been reported in other studies, in contrast to the current study (preterm rate of 5.8%). The authors speculate that this could probably be because of the fact that the pregnant women in this region are healthy. They are young, do not smoke or drink alcohol, and have very few or nil preexisting illnesses. They avail antenatal services regularly and institutional deliveries are the norm.

## 5. Conclusion 

The commonest obstetrical risk factor for preterm birth was hypertensive disorders of pregnancy and nonobstetrical risk factor was height <1.50 m. Significant risk factors for preterm birth found in this study were gestational hypertension, height <1.50 m, preterm rupture of membranes, threatened abortion, and twin gestation.

## Figures and Tables

**Table 1:** Diagnostic criteria for clinical conditions.

Clinical condition	Criteria
Gestational diabetes mellitus	Fasting plasma glucose ≥92 mg/dL to 126 mg/dL (as fasting plasma glucose ≥126 mg/dL is consistent with overt diabetes)

Pregnancy induced hypertension	Systolic blood pressure ≥130 mm Hg and/or Diastolic blood pressure ≥90 mm Hg

Anemia	Hemoglobin <11.0 g/dL

Oligohydramnios	Amniotic fluid index <8

**Table 2:** Characteristics of the preterm and term deliveries.

	Cases (*n* = 154)		Controls (*n* = 334)	
	Mean	Standard deviation	Mean	Standard deviation
Age in years	27.5	3.97	27.1	3.62
Height (cm)	155.4	5.99	156.1	5.35
Weight (kg)	61.0	13.00	60.5	10.35
Birth weight (gm)	2452.7	436.01	2977.8	401.97
Antenatal check-ups (number)	6.9	4.12	8.5	3.89
Hemoglobin (g/dL)	11.8	1.27	12.1	1.95

**Table 3:** Mode of delivery of study population.

Mode of delivery	Cases (*n* = 152) *N* (%)	Controls (*n* = 334) *N* (%)	*P* value	Odds ratio	95% CI
Spontaneous vaginal delivery	63 (41.4)	171 (51.2)	**0.03**	0.66	**0.45–0.97**
Assisted vaginal delivery	09 (5.9)	34 (10.2)	0.12	0.55	0.26–1.17
Elective LSCS*	18 (11.8)	58 (17.4)	0.11	0.63	0.36–1.01
Emergency LSCS*	62 (40.8)	71 (21.3)	**<0.001**	2.50	**1.65–3.78**

*LSCS: lower segment caesarean section.

**Table 4:** Preexisting medical conditions among the mothers.

Variables	Cases (*n* = 154) *N* (%)	Controls (*n* = 334) *N* (%)	Odds ratio	95% CI
Preexisting medical conditions				
Diabetes	01 (0.7)	0	—	—
Hypertension	01 (0.7)	01 (0.0)	2.18	0.14–35.03
Asthma or bronchitis	03 (1.9)	06 (1.8)	1.09	0.27–4.40
Thyroid disorder	03 (1.9)	05 (1.5)	1.31	0.31–5.54

Family history				
Diabetes	19 (12.3)	38 (11.4)	1.10	0.61–1.97
Hypertension	21 (13.6)	54 (16.1)	0.82	0.48–1.41

Complications during previous pregnancies				
Pregnancy induced hypertension	08 (5.2)	04 (1.2)	4.52	1.34–15.25
Gestational diabetes mellitus	01 (0.7)	02 (0.6)	1.09	0.10–12.06
Oligohydramnios	01 (0.7)	03 (0.9)	0.72	0.07–6.99
Cephalopelvic disproportion	03 (1.9)	06 (1.8)	1.09	0.27–4.40
Failed induction	01 (0.7)	11 (3.3)	0.19	0.03–1.50
Fetal distress	03 (1.9)	08 (2.4)	0.81	0.21–3.10
Premature rupture of membranes	0	01 (0.3)	—	—
Placenta previa	05 (3.2)	01 (0.3)	11.17	1.29–96.48

**Table 5:** Risk factors associated with preterm birth.

Risk factors	Cases (*n* = 154) *N* (%)	Controls (*n* = 334) *N* (%)	*P* value	Odds ratio (95% CI)
Gestational hypertension	33 (21.4)	26 (7.8)	**<0.001**	**3.23 (1.85–5.63)**
Height <1.50 m	26 (16.8)	32 (9.6)	**0.02**	**1.96 (1.12–3.42)**
Preterm rupture of membranes (PROM)	27 (17.5)	21 (6.3)	**<0.001**	**3.17 (1.73–5.81)**
Fetal distress	23 (14.9)	53 (15.9)	0.79	0.93 (0.55–1.59)
Oligo-/polyhydramnios	19 (12.3)	23 (6.9)	**0.046**	1.90 (1.00–3.61)
Uterine scar	16 (10.4)	27 (8.1)	0.40	1.32 (0.69–2.52)
Infection	15 (9.7)	25 (7.5)	0.40	1.33 (0.68–2.61)
Threatened abortion	14 (9.1)	11 (3.3)	**<0.01**	**2.94 (1.30–6.63)**
Failed induction	14 (9.1)	45 (13.5)	0.17	0.64 (0.34–1.21)
Twin gestation	13 (8.4)	04 (1.2)	**<0.001**	**7.60 (2.44–23.73)**
Gestational diabetes mellitus (GDM)	13 (8.4)	27 (8.1)	0.90	1.05 (0.53–2.09)
Malpresentation	13 (8.4)	14 (4.2)	0.06	2.10 (0.97–4.60)
Threatened preterm	08 (5.2)	07 (2.1)	0.09	2.56 (0.91–7.91)
Maternal disease	05 (3.3)	06 (1.8)	0.32	1.83 (0.55–6.11)
Hyperemesis gravidarum	05 (3.3)	10 (3.0)	0.88	1.09 (0.37–3.24)
Cephalopelvic disproportion	03 (2.0)	13 (3.9)	0.26	0.49 (0.14–1.75)
Antepartum haemorrhage (APH)	05 (3.3)	04 (1.2)	0.12	2.77 (0.73–10.46)
Anemia	03 (2.0)	05 (2.0)	0.72	1.31 (0.31–5.54)
Others*	0	07 (2.1)	—	—

*Safe confinement, baby hydrocephalus with absent posterior vault, bladder injury, and fetal polycystic kidneys.
